# Optimization of glycerol and egg yolk concentrations for cryopreservation of Saanen buck sperm under tropical conditions in the Mekong Delta, Vietnam

**DOI:** 10.14202/vetworld.2025.3631-3639

**Published:** 2025-11-29

**Authors:** Duy Lam Khanh Nguyen, Hien Thi Dieu Nguyen, Khuong Thi Thanh Tran

**Affiliations:** 1 Graduate School, Can Tho University, Can Tho City, Vietnam; 2 Stem Cell Laboratory, Institute of Food and Biotechnology, Can Tho University, Can Tho City, Vietnam

**Keywords:** Cryopreservation, DNA fragmentation, egg yolk, glycerol, Saanen buck, sperm viability, tropical conditions

## Abstract

**Background and Aim:**

Sperm cryopreservation is a cornerstone technology for genetic resource conservation and artificial insemination. However, tropical climatic conditions often compromise post-thaw sperm quality due to enhanced oxidative and thermal stress. This study aimed to optimize glycerol and egg yolk concentrations in a Tris–citrate–glucose (TCG) extender to improve post-thaw sperm quality of Saanen bucks raised in the Mekong Delta, Vietnam.

**Materials and Methods:**

The experiment was conducted in two sequential phases. In Phase 1, five glycerol concentrations (4%, 6%, 8%, 10%, and 12%) were tested to identify the optimal permeating cryoprotectant level. In Phase 2, four egg yolk concentrations (5%, 10%, 15%, and 20%) were assessed in combination with the optimal glycerol concentration. Semen from four healthy Saanen bucks was collected using an artificial vagina, diluted in the respective extenders, equilibrated, and frozen in liquid nitrogen. Post-thaw evaluations included overall and progressive motility (phase-contrast microscopy), viability (eosin–nigrosin staining), membrane integrity (hypo-osmotic swelling test), acrosome integrity (Giemsa staining), and DNA fragmentation (sperm chromatin dispersion test). Data were analyzed using one-way analysis of variance with Tukey’s post hoc test at p < 0.05.

**Results:**

Glycerol concentration had a significant effect on post-thaw sperm quality (eta squared = 0.93–0.97), with 10% providing the best balance between cryoprotection and cytotoxicity. Incorporating 15% egg yolk with 10% glycerol significantly improved sperm viability (65.5%), membrane integrity (72.3%), and reduced DNA fragmentation (12.4%) compared with other treatments (p < 0.05). Higher glycerol or egg yolk concentrations adversely affected motility due to increased osmotic stress and viscosity.

**Conclusion:**

The combination of 10% glycerol and 15% egg yolk in TCG extender provides optimal cryoprotection for Saanen buck semen under tropical conditions. The resulting post-thaw sperm exhibited high motility, viability, and DNA integrity. This protocol can serve as a region-specific standard for buck semen cryobanking and artificial insemination in tropical climates, supporting genetic improvement and conservation initiatives in Vietnam and other developing regions.

## INTRODUCTION

Sperm cryopreservation remains a cornerstone of reproductive biotechnology, serving as an indispensable tool for artificial insemination, gene banking, and the preservation of valuable animal genetic resources [1]. Nevertheless, the freeze–thaw process subjects spermatozoa to a range of biophysical and biochemical stressors, such as cold shock, osmotic imbalance, intracellular ice crystal formation, and oxidative injury, that collectively compromise membrane integrity, mitochondrial function, and fertilizing potential [2]. To counteract these effects, cryoprotective agents (CPAs) are incorporated into semen extenders to stabilize sperm structure and function during the freezing process. Among these, glycerol, a permeating CPA, plays a critical role in reducing intracellular ice crystal formation by replacing water molecules and stabilizing membrane phospholipids [3]. In contrast, egg yolk functions as a non-permeating CPA that offers extracellular protection through its low-density lipoproteins (LDLs) and phospholipids, which safeguard the sperm plasma membrane against lipid peroxidation and thermal stress [4]. The combined use of glycerol and egg yolk has been shown to enhance post-thaw sperm survival across several species, including buffalo [5], Boer goats [6], and rabbits [7].

However, the ideal concentrations of these cryoprotectants differ depending on species, breed, and environmental factors. Excessive glycerol may induce cytotoxicity, disrupt mitochondrial respiration, and alter chromatin configuration, whereas an overly concentrated egg yolk can elevate medium viscosity, hinder sperm respiration, and impair motility [8]. Furthermore, many cryopreservation protocols are designed for temperate climates and may not perform optimally in tropical regions, where elevated temperatures and oxidative stress intensify cellular damage [9, 10].

In Vietnam, goat production plays a vital role in food security and rural economic resilience but faces ongoing challenges from disease outbreaks and climate-related stresses [11]. Rapid genetic restoration and productivity enhancement are, therefore, national priorities, for which semen cryopreservation provides a practical and sustainable solution. The Saanen goat (*Capra hircus*), a globally recognized dairy breed known for its high milk yield and adaptability, is gaining importance in tropical farming systems. Yet, most existing cryopreservation protocols have been formulated under temperate conditions, leaving limited data for Saanen goats raised in tropical environments. Hence, there is a compelling need to develop an optimized, climate-adapted cryopreservation protocol for Saanen buck semen to strengthen artificial insemination programs and genetic conservation of small ruminants in Vietnam [7, 12].

Although cryopreservation techniques and extender formulations have been widely optimized for cattle, buffalo, and temperate goat breeds, the available data on Saanen buck semen cryopreservation under tropical climatic conditions remain scarce and fragmented. Most established protocols were designed for animals maintained in temperate or controlled environments, where ambient temperature and oxidative load are markedly lower than in tropical systems. Consequently, their direct adoption in regions such as the Mekong Delta often results in suboptimal post-thaw motility, viability, and DNA integrity, primarily due to heat-induced oxidative stress and osmotic instability. Furthermore, studies investigating the interactive effects of permeating (glycerol) and non-permeating (egg yolk) cryoprotectants in tropical buck semen have been limited. Existing literature typically reports results for a single cryoprotectant variable or uses formulations optimized for other species, such as buffalo or Boer goats [5–7]. There is also a lack of quantitative evaluation of DNA fragmentation and membrane integrity under tropical storage conditions—critical parameters for assessing true sperm functionality beyond motility alone. Thus, no comprehensive, climate-specific optimization has been developed for Saanen buck semen cryopreservation in Vietnam or comparable tropical regions. Addressing this gap is essential to enable the establishment of goat cryobanks and ensure the long-term genetic sustainability of high-yield dairy breeds adapted to smallholder systems.

This study was designed to develop and optimize a region-specific cryopreservation protocol for Saanen buck semen tailored to the tropical environmental conditions of the Mekong Delta, Vietnam. The research specifically aimed to (i) determine the optimal concentration of glycerol as a permeating cryoprotectant to minimize intracellular ice formation and osmotic stress, and (ii) identify the appropriate proportion of egg yolk as a non-permeating cryoprotectant to maximize extracellular membrane protection and post-thaw sperm integrity. A two-phase experimental design was employed: first, varying glycerol concentrations (4%–12%) were tested; then, different egg yolk concentrations (5%–20%) were combined with the optimal glycerol level to comprehensively evaluate the effects on motility, viability, membrane integrity, acrosome status, and DNA fragmentation. By integrating these quality indicators, the study aimed to establish a scientifically validated, cost-effective, and climate-resilient cryopreservation protocol suitable for artificial insemination and the conservation of genetic resources in Saanen bucks in tropical small ruminant production systems.

## MATERIALS AND METHODS

### Ethical approval

All animal procedures were conducted in accordance with the ethical standards for animal research of Can Tho University. The experimental protocol, including animal handling, feeding, and semen collection, was reviewed and approved by the Animal Experiments Ethics Committee of Can Tho University, Vietnam (Approval ID: CTU-AEC24013). The study adhered to the Animal Research: Reporting of *In Vivo* Experiments guidelines and followed national regulations on the care and use of laboratory animals. Efforts were made to minimize animal discomfort and to ensure proper housing, nutrition, and environmental enrichment throughout the study.

### Study period and location

The experiment was carried out from July to December 2024 at the same facility in the Mekong Delta, Vietnam (10°01′N, 105°45′E). This period corresponds to the rainy season, with an average temperature of 29°C–31°C and relative humidity of 70%–80%. Frequent rainfall during this season enhances forage availability and reproductive performance, creating suitable environmental conditions for assessing semen cryopreservation efficiency under realistic tropical settings.

### Animals

Four healthy 2-year-old Saanen bucks (40–45 kg) were maintained at the experimental animal farm of the Stem Cell Laboratory, Institute of Biotechnology and Food Technology, Can Tho University (10°01′N, 105°45′E). The bucks were housed individually in well-ventilated pens (1.8 m × 1.5 m × 2.0 m) under hygienic conditions. The average ambient temperature during the study period was 30°C ± 2°C with a relative humidity of 75% ± 5%. The Mekong Delta region experiences a tropical monsoon climate characterized by two distinct seasons: the dry season (December–April) and the rainy season (May–November), which influence natural breeding activity in goats. Feed was formulated according to the National Research Council guidelines [13], and water was provided *ad libitum*.

### Preparation of extenders

#### Base Tris–Citrate–Glucose (TCG) extender

The TCG extender was prepared in double-distilled water containing 250 mmol/L Tris-hydroxymethylaminomethane (BioBasic, Canada), 88 mmol/L citric acid (Sigma-Aldrich, USA), 47 mmol/L D-glucose (Thermo Fisher Scientific, USA), and 80 mg/L gentamicin sulfate. The pH was adjusted to a range of 6.9–7.1.

#### Glycerol treatments

Five glycerol concentrations (4%, 6%, 8%, 10%, and 12% v/v) were tested in the base extender to determine the optimal permeating cryoprotectant level.

#### Egg yolk preparation and concentrations

Fresh chicken eggs (Ba Huân Company, Vietnam) were manually separated, and yolks were homogenized and filtered through sterile gauze. The filtered yolk was added to extenders at 5%, 10%, 15%, and 20% (v/v) after establishing the optimal glycerol concentration.

### Experimental design

The experiment comprised two sequential phases within a single design framework:

- Phase 1: Evaluation of six glycerol concentrations (0% – control, 4%, 6%, 8%, 10%, and 12% v/v) in the TCG extender to determine the optimal concentration for Saanen buck sperm cryopreservation.
- Phase 2: Assessment of five egg yolk concentrations (0% – control, 5%, 10%, 15%, and 20% v/v) combined with the optimal glycerol level identified in Phase 1.

### Semen collection and evaluation

Each buck was sexually rested for 7 days before semen collection. Ejaculates were collected twice weekly using an artificial vagina pre-warmed to 38°C, yielding six ejaculates per buck. After centrifugation to remove seminal plasma, samples were pooled to minimize individual variation. Ejaculates meeting the following criteria were included: volume ≥0.5 mL, pH 6.8–7.2, motility ≥70%, morphology ≥70%, and viability ≥80%.

### Cryopreservation procedures

#### Dilution and equilibration

Semen was diluted to a final concentration of 2 × 10^8^ sperm/mL with the respective extenders, mixed gently, and loaded into 0.5 mL straws.

#### Cooling and freezing

To minimize thermal shock, samples were cooled in two stages: 15°C for 30 min, followed by 5°C for 60 min (cooling rate ≈ 0.2°C/min). The straws were then exposed to liquid nitrogen vapor (≈ 5 cm above LN_2_ surface) for 15 min before complete immersion in LN_2_ for long-term storage [14].

#### Thawing

After 72 h of storage, the straws were thawed in a 37°C water bath for 30 s, and semen was transferred into 2 mL centrifuge tubes for post-thaw analysis.

### Post-Thaw quality assessment

#### Motility (phase-contrast microscopy)

A 10 μL aliquot was examined under a phase-contrast microscope (Nikon Eclipse, Japan, 400×). Overall and progressive motility were assessed visually in five random fields per sample [15].

#### Viability (Eosin–Nigrosin staining)

**Figure 1:**
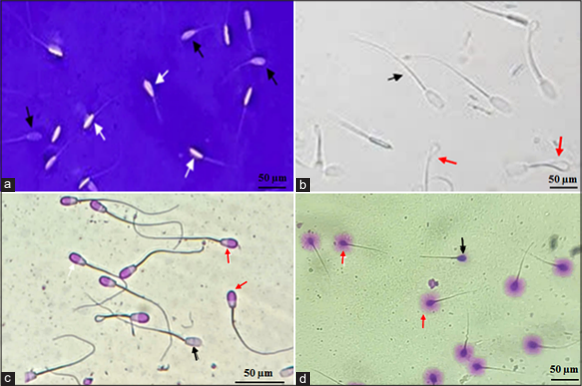
Representative photomicrographs of post-thaw Saanen buck sperm quality parameters. (a) Eosin–nigrosin staining: unstained live cells (white arrows) and stained dead cells (pink arrows). (b) Hypo-osmotic swelling test: Intact membranes show tail coiling (red arrows), whereas damaged membranes show straight tails (black arrow). (c) Giemsa staining of acrosomes: intact acrosomes stain pink (red arrows), whereas disrupted acrosomes remain unstained (black arrow). (d) Sperm chromatin dispersion test: intact DNA has halos (red arrows) and fragmented DNA has no halos (black arrow). Scale bar = 50 μm.

Equal volumes (10 μL) of semen and eosin–nigrosin solution were mixed, smeared on a slide, and examined at 400×. Live sperm appeared unstained, whereas dead sperm stained purple [16]. Representative images are shown in Figure 1a.

#### Membrane integrity (hypo-osmotic swelling test [HOST])

The HOST was performed using a 100 mOsm/kg solution following Tran *et al*. [17]. Sperm with coiled tails were considered membrane-intact (Figure 1b).

#### Acrosome integrity (Giemsa staining)

Samples (20 μL) were air-dried, fixed in 96% ethanol for 15 s, and stained with 10% Giemsa solution for 10 min, following Vietnamese Ministry of Health guidelines [18]. Intact acrosomes appeared as dark crescent caps (Figure 1c).

#### DNA fragmentation (sperm chromatin dispersion [SCD] test)

DNA integrity was assessed using the SCD test [19]. Sperm were sequentially treated with 0.15 mol/L hydrochloric acid (15 min, dark) and 30 mol/L dithiothreitol (30 min), followed by 30% Giemsa staining. Sperm with intact DNA exhibited large halos, whereas fragmented DNA showed small or no halos (Figure 1d).

### Statistical analysis

Data were analyzed to evaluate the effects of glycerol and egg yolk concentrations on post-thaw semen quality. Normality and variance homogeneity were verified using the Shapiro–Wilk and Levene tests, respectively. One-way analysis of variance followed by Tukey’s *post hoc* test was applied (p < 0.05). Analyses were performed in R v4.3.1 (http://www.R-project.org/) using the *agricolae*, *ggplot2*, and *ggpubr* packages. Biological replicates included semen from four bucks (three ejaculates each; n = 12) with three technical fields per sample. Data are presented as mean ± standard error, and eta squared (η²) values were calculated to estimate treatment effect size.

## RESULTS

### Effect of glycerol concentration on post-thaw sperm quality

**Figure 2:**
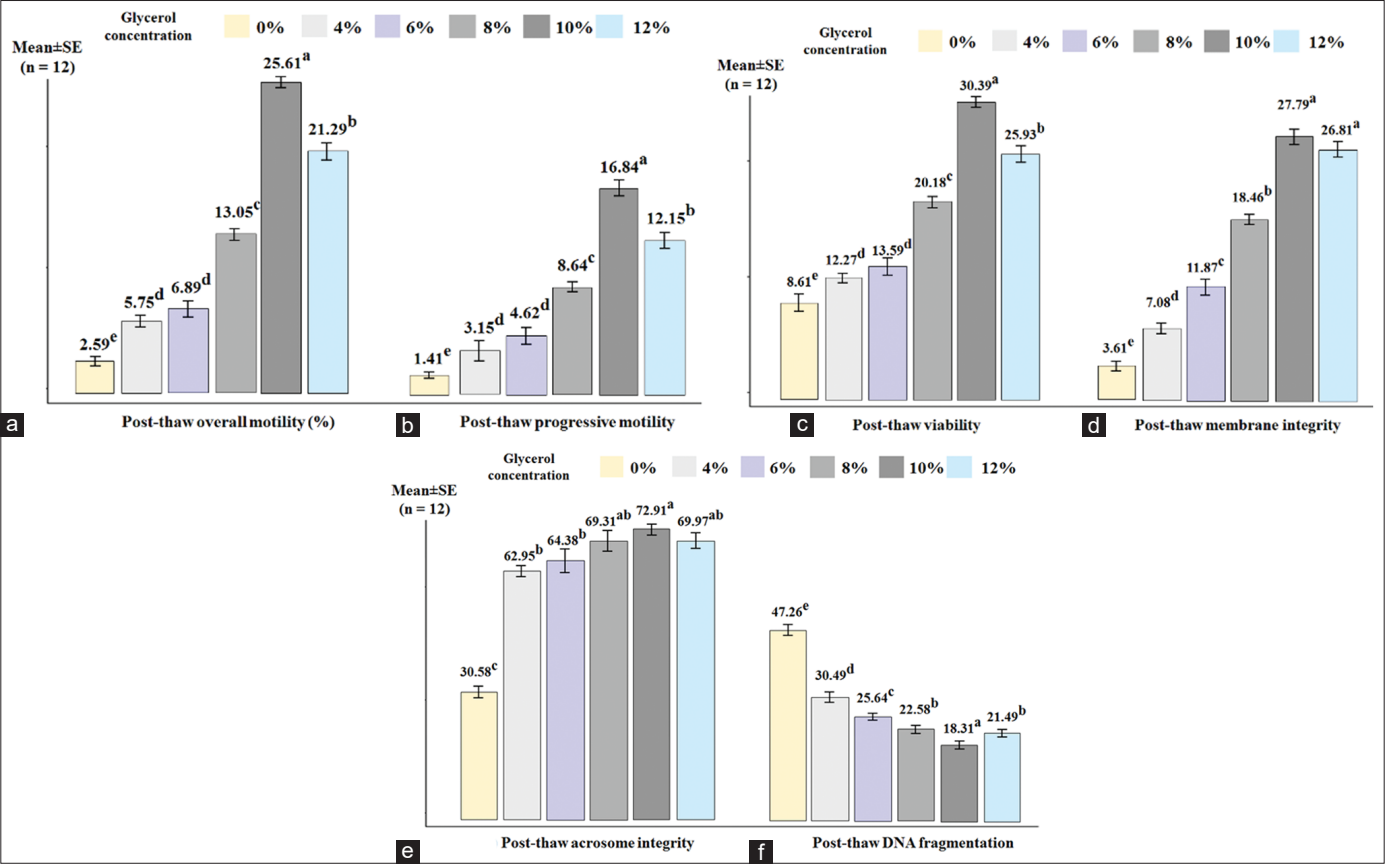
Post-thaw sperm quality parameters of Saanen buck semen at different concentrations of glycerol (0%, 4%, 6%, 8%, 10%, and 12%). Bars represent mean ± standard error (n = 12). Different glycerol concentrations significantly influenced multiple quality indicators. Each subplot corresponds to a specific parameter: (a) Overall motility, (b) progressive motility, (c) viability, (d) membrane integrity, (e) acrosome integrity, and (f) DNA fragmentation. ^a,b,c,d,e^Values for each data in one criterion with different superscripts are statistically significantly different; p < 0.05.

Glycerol concentration significantly influenced post-thaw sperm quality in Saanen bucks, as illustrated in Figure 2. Data analysis showed that glycerol concentration had a strong impact on sperm quality parameters, such as overall motility (η² = 0.954), progressive motility (η² = 0.937), viability (η² = 0.951), membrane integrity (η² = 0.947), acrosome integrity (η² = 0.970), and DNA fragmentation (η² = 0.951).

The evaluation results showed that 10% glycerol significantly improved sperm quality (p < 0.05) in terms of overall motility, progressive motility, viability, and DNA fragmentation. Regarding acrosome integrity, the 10% glycerol concentration was not significantly different from the 8% and 12% glycerol concentrations (p > 0.05). Similarly, the sperm membrane integrity was not significantly different between the 10% and 12% glycerol concentrations (p > 0.05).

### Effect of egg yolk concentration on post-thaw Saanen buck sperm quality

**Figure 3:**
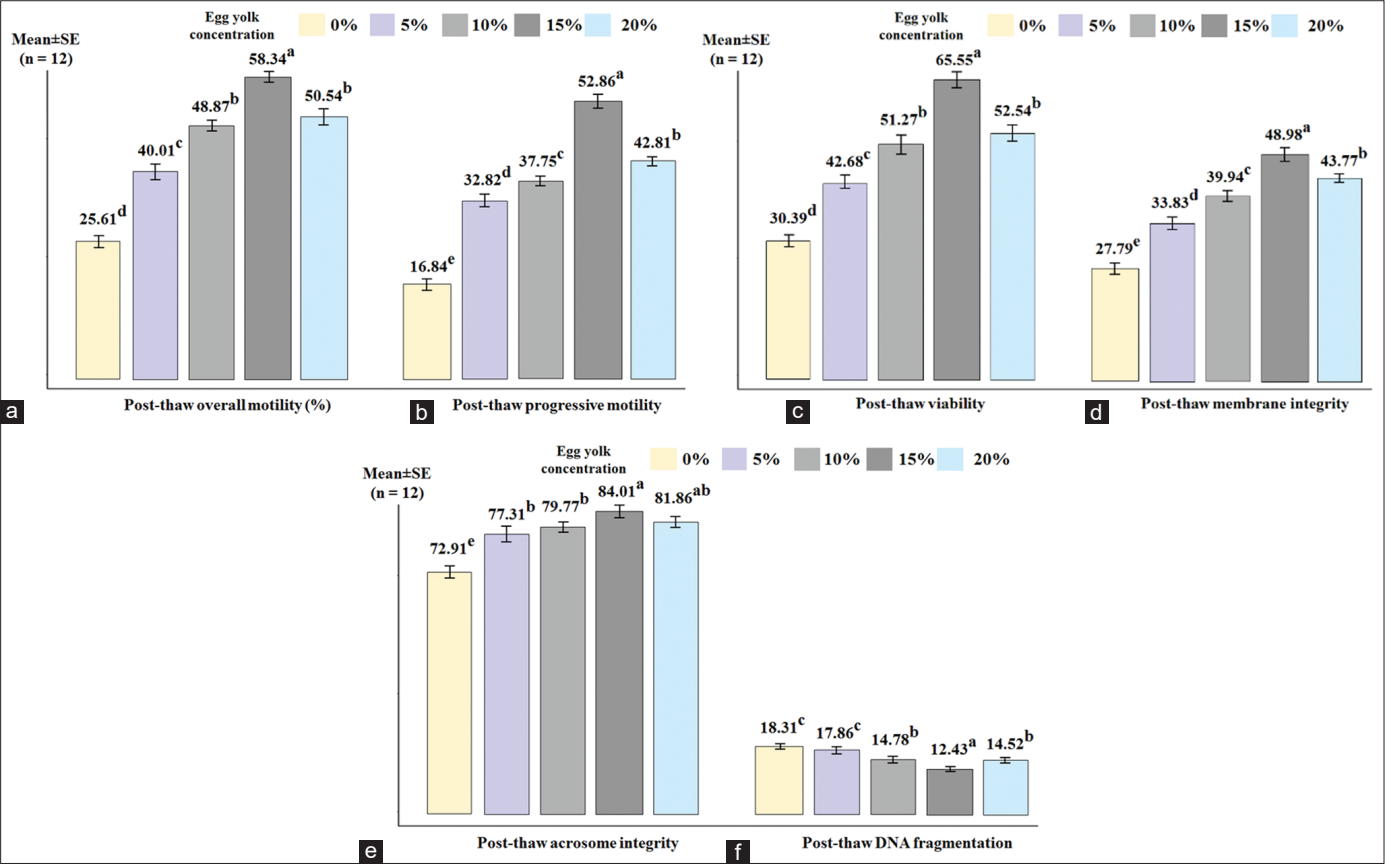
Post-thaw sperm quality parameters of Saanen buck semen at different egg yolk concentrations (0%, 5%, 10%, 15%, and 20%). Bars represent mean ± standard error (n = 12). Different egg yolk concentrations significantly influenced multiple quality indicators. Each subplot corresponds to a specific parameter: (a) Overall motility, (b) progressive motility, (c) viability, (d) membrane integrity, (e) acrosome integrity, and (f) DNA fragmentation. ^a,b,c,d,e^Values for each data in one criterion with different superscripts are statistically significant differences; p < 0.05.

The concentration of egg yolk in the cryopreservation extender significantly influenced post-thaw sperm quality, as shown in Figure 3. Similar to glycerol, egg yolk also had a strong effect on sperm quality parameters such as overall motility (η² = 0.988), progressive motility (η² = 0.987), viability (η² = 0.993), membrane integrity (η² = 0.960), acrosome integrity (η² = 0.758), and DNA fragmentation (η² = 0.816).

The combination of 10% glycerol and egg yolk improved sperm quality better than glycerol alone. The 15% egg yolk concentration significantly improved sperm quality in terms of overall motility, progressive motility, viability, membrane integrity, and DNA fragmentation (p < 0.05). However, the sperm acrosome integrity was not significantly different between the 15% and 20% egg yolk concentrations (p > 0.05).

## DISCUSSION

### Optimization of glycerol concentration for cryopreservation

The results of phase 1 showed that a 10% glycerol concentration was optimal for the cryopreservation of Saanen buck sperm, while our other study on Boer bucks suggested that 8% glycerol was the most effective concentration [6]. In phase 2, an egg yolk concentration above 10% helped improve sperm motility; this result is similar to that of Sharma *et al*. [20]. A 15% chicken egg yolk concentration yielded an overall motile sperm rate of 58.34% and a progressive motility rate of 52.86%. The best overall and progressive sperm motility rates in the study by Aboagla and Terada [21] were 59% and 46%, respectively, when using a 20% egg yolk concentration.

During cold storage, glycerol helps lower the freezing point and reduce ice crystal formation in cells by penetrating the cell membrane, stabilizing the lipid bilayer, and binding to intracellular water [22]. However, the results showed that when excessively high concentrations of glycerol were added, its strong activity negatively affected the structure of the cell membrane. According to Donnelly *et al*. [8], the decrease in sperm motility after thawing was attributed to mitochondrial damage during cryopreservation, along with a decrease in intracellular ATP concentration and a significant increase in ROS levels.

Glycerol plays a dual role in sperm cryopreservation: it protects cells from ice crystal formation but can also negatively affect mitochondrial function. High glycerol concentrations may induce mitochondrial swelling, disrupt mitochondrial membrane potential, and impair ATP production, ultimately reducing sperm motility and viability [23]. Therefore, optimizing glycerol levels is essential to balance its cryoprotective benefits with its potential mitochondrial toxicity. Glycerol concentration affects sperm quality differently depending on the animal species. For example, Tran *et al*. [7] reported that a concentration of 5% glycerol was more effective than 8% for the cryopreservation of rabbit sperm.

### Protective role of egg yolk as a non-permeating cryoprotectant

Egg yolk is the main non-permeating cryoprotectant used in semen preservation media and protects sperm from thermal shock during the freezing process [24]. Egg yolk–supplemented storage media are commonly used in semen cryopreservation because egg yolk prevents membrane phospholipid loss during freezing [25]. LDL from egg yolk plays a crucial role in spermatozoa protection during cooling and cryopreservation. LDL forms a protective layer around the sperm plasma membrane, helping stabilize the membrane structure, reducing the loss of phospholipids and cholesterol, and preventing the formation of ice crystals that can damage the cell. Moreover, LDL minimizes enzyme and ion leakage, maintains mitochondrial function, and preserves membrane integrity, ultimately improving post-thaw motility and viability [26].

### Combined effect of glycerol and egg yolk

Our findings demonstrated that using a diluent containing 10% glycerol and 15% egg yolk provided the best protection for Saanen buck semen during cryopreservation, resulting in superior post-thaw motility and viability. In contrast, Nisfimawardah *et al*. [27] reported no significant differences in semen quality when using tris–egg yolk, AndroMed, or OviXcell diluents. This contrast highlights the critical role of optimizing glycerol and egg yolk concentrations in sperm integrity preservation during freezing.

Bogdaniuk and Petrushko used 10% glycerol + 20% egg yolk and focused on seasonal effects on sperm morphometry, reporting significant post-thaw morphological changes [28]. This indicates that although both studies used glycerol–egg yolk diluents, ours emphasized functional quality, whereas theirs focused on structural alterations. Suwor *et al*. [29] tested various cryoprotectant combinations in Boer bucks and found that 5% glycerol + 18% egg yolk yielded the best results in motility, biological function, and pregnancy rate (66.67%), outperforming the control.

### Novelty and regional significance of the study

To the best of our knowledge, this is the first systematic optimization of glycerol and egg yolk concentrations for Saanen buck semen cryopreservation in the Mekong Delta under tropical conditions. Most existing cryopreservation protocols were developed for temperate climates such as Europe and North America, where oxidative stress is lower and temperatures are milder. Their direct application in tropical regions often results in suboptimal post-thaw sperm quality. This study bridges this gap by developing a region-specific protocol that addresses the thermal and oxidative challenges unique to tropical systems.

Saanen goats are among the world’s most important dairy breeds; however, studies on semen preservation remain limited compared to Boer or indigenous breeds. The optimized extender identified in this study provides a breed-specific and climate-adapted approach that can directly improve artificial insemination efficiency and dairy goat breeding in tropical Asia. Moreover, this study incorporated SCD to evaluate DNA fragmentation, a methodological advance in semen cryobiology, unlike previous research that focused mainly on motility and viability.

### Practical implications and one health relevance

Beyond laboratory relevance, these findings establish a foundation for genetic resource banking and AI programs in developing tropical regions, contributing to food security, livestock improvement, and genetic conservation. This work also aligns with climate adaptation and One Health frameworks, as cryobanking resilient germplasm supports the recovery of goat populations under threats such as heat stress, foot-and-mouth disease, and peste des petits ruminants. Our results achieved comparable or superior post-thaw motility and DNA integrity compared with reported values in Boer goats, buffalo, rabbits, and stallions, underscoring the novelty and robustness of this tropical-specific protocol for dairy goat genetic resource preservation.

### Limitations and future research directions

This study focused on optimizing glycerol and egg yolk concentrations in a TCG extender for Saanen buck semen cryopreservation under tropical conditions. However, the absence of antioxidant supplementation and comparison with commercial extenders should be acknowledged as limitations. Antioxidants, such as Vitamin E, selenium, or plant-derived polyphenols, may further reduce oxidative stress and improve post-thaw sperm quality, warranting inclusion in future formulations.

In addition, the current findings are based solely on *in vitro* sperm parameters (motility, viability, membrane integrity, acrosome status, and DNA integrity). Future research should include artificial insemination trials to assess fertility outcomes such as conception, pregnancy, and kidding rates to validate the field applicability of the optimized extender. These subsequent studies will provide stronger evidence for the large-scale implementation of cryopreserved Saanen buck semen in tropical breeding programs.

## CONCLUSION

The present study successfully optimized a cryopreservation protocol for Saanen buck semen under tropical conditions by adjusting the concentrations of permeating and non-permeating cryoprotectants. The findings demonstrated that a TCG extender supplemented with 10% glycerol and 15% egg yolk provided the most effective cryoprotection, yielding the highest post-thaw sperm motility, viability, membrane and acrosome integrity, and the lowest DNA fragmentation rate. These results confirm that an appropriate balance between glycerol and egg yolk concentrations is critical to mitigating cryo-injury and preserving mitochondrial and genomic stability in buck spermatozoa.

A key strength of this study lies in its region-specific optimization under real tropical conditions, representing the first systematic attempt to refine Saanen buck semen cryopreservation for the Mekong Delta. The use of multiple complementary assays, motility, viability, membrane integrity, acrosomal status, and DNA fragmentation, ensured a comprehensive assessment of post-thaw sperm quality. Moreover, by integrating quantitative indicators, such as η² effect sizes, this study provides reproducible, statistically supported evidence of treatment efficacy.

Overall, the optimized combination of 10% glycerol and 15% egg yolk offers a cost-effective, reproducible, and climate-adapted protocol suitable for artificial insemination programs and genetic conservation of dairy goats in tropical regions. This protocol contributes to the establishment of regional cryobanks and supports livestock improvement strategies aligned with climate resilience and One Health goals. Future studies should include *in vivo* fertility trials and explore the addition of antioxidants or natural cryoprotectants to further enhance post-thaw sperm performance and validate the field applicability of this tropical-specific cryopreservation system.

## DATA AVAILABILITY

The supplementary data can be made available from the corresponding author upon request.

## AUTHORS’ CONTRIBUTIONS

DLKN: Contributed to the development of the methodology, statistical analysis, and data validation, and participated in manuscript writing and revision. HTDN: Conducted the experiments, collected samples, and prepared data visualizations. KTTT: Conceptualized and supervised the research, coordinated the research activities, contributed to the experimental design, and led the final manuscript review and editing. All authors have read and approved the final manuscript.

## References

[1] Engdawork A, Belayhun T, Aseged T (2024). The role of reproductive technologies and cryopreservation of genetic materials in the conservation of animal genetic resources. Ecol. Genet. Genomics.

[2] Kumar A, Prasad J.K, Srivastava N, Ghosh S.K (2019). Strategies to minimize various stress-related freeze-thaw damages during conventional cryopreservation of mammalian spermatozoa. Biopreserv. Biobank.

[3] Ma L, Kim D.H, Jung E.J, Lee W.J, Hwang J.M, Bae J.W, Jung D.J, Yi J.K, Lee S.M, Ha J.J, Kwon W.S (2022). Effect of glycerol addition time on the cryopreserved Korean native brindle cattle (Chikso) sperm quality. Anim. Reprod.

[4] Amirat L, Anton M, Tainturier D, Chatagnon G, Battut I, Courtens J.L (2005). Modifications of bull spermatozoa induced by three extenders:Biociphos, low density lipoprotein and Triladyl, before, during and after freezing and thawing. Reproduction.

[5] El-Sisy G.A, Shahba M.I, El-Sheshtawy R.I (2016). Freezability of buffalo semen with TRIS extender enriched with disaccharides (trehalose or sucrose) and different glycerol concentrations. Asian Pac. J. Reprod.

[6] Tran T.T.T, Nguyen L.K.D, Nguyen T.K.N, Tran V.B.N, Duong N.D.T, Lam P.T (2024). Cryopreservation of goat spermatozoa in the Mekong Delta region of Vietnam:Evaluating cryoprotectant effectiveness. Vet. Integr. Sci.

[7] Tran T.T.K, Chiem T.N, Tran V.B.N, Duong N.D.T (2023). Cryopreservation of local black rabbit spermatozoa in the Mekong delta region of Vietnam:Evaluating cryoprotectant effectiveness. Reprod. Domest. Anim.

[8] Donnelly E.T, McClure N, Lewis S.E (2001). Cryopreservation of human semen and prepared sperm:Effects on motility parameters and DNA integrity. Fertil. Steril.

[9] Zhong R.Z, Zhou D.W (2013). Oxidative stress and role of natural plant derived antioxidants in animal reproduction. J. Integr. Agric.

[10] Contreras M.J, Treulen F, Arias M.E, Silva M, Fuentes F, Cabrera P, Felmer R (2020). Cryopreservation of stallion semen:Effect of adding antioxidants to the freezing medium on sperm physiology. Reprod. Domest. Anim.

[11] Tran T.T.K, Lam P.T, Nguyen T.K.K, Nguyen T.N, Van Duong N.D.T (2022). Cryobank:A rapid recovery solution for livestock populations after disease outbreaks. Can Tho University J. Sci.

[12] Hegde N.G (2020). Goat development:An opportunity to strengthen rural economy in Asia and Africa. Asian J. Res. Anim. Vet. Sci.

[13] NRC (2007). Nutrient Requirements of Small Ruminants.

[14] Khuong T.T.T, Duy N.L.K, Ngan N.T.K, Nam T.V.B, Tuyen D.N.D, Thanh L.P (2024). Cryopreservation of goat spermatozoa in the Mekong Delta region of Vietnam:Evaluating cryoprotectant effectiveness. Vet. Integr. Sci.

[15] Fumuso F.G, Giuliano S.M, Chaves M.G, Neild D.M, Miragaya M.H, Gambarotta M.C, Carretero M.I (2018). Seminal plasma affects the survival rate and motility pattern of raw llama spermatozoa. Anim. Reprod. Sci.

[16] Boccia L, Di Palo R, De Rosa A, Attanasio L, Mariotti E, Gasparrini B (2007). Evaluation of buffalo semen by Trypan blue/Giemsa staining and related fertility *in vitro*. Ital. J. Anim. Sci.

[17] Tran K.T.T, Nguyen T.N, Nguyen D.L.K (2025). Developing a simple universal hypo-osmotic swelling test (HOST) for assessing sperm membrane integrity in pigs, rabbits, and goats. J. Adv. Vet. Anim. Res.

[18] Vietnamese Ministry of Health (2016). Guidance on Technical Procedures for Pathological Anatomy and Cytology.

[19] Absalan F, Ghannadi A, Kazerooni M, Parifar R, Jamalzadeh F, Amiri S (2012). Value of sperm chromatin dispersion test in couples with unexplained recurrent abortion. J. Assist. Reprod. Genet.

[20] Sharma A, Sood P, Chaudhary J.K (2020). Comparative efficacy of different concentrations of egg yolk for cryopreservation of goat semen. Indian J. Anim. Sci.

[21] Aboagla E.M.E, Terada T (2004). Effects of egg yolk during the freezing step of cryopreservation on the viability of goat spermatozoa. Theriogenology.

[22] Bakhach J (2009). The cryopreservation of composite tissues:Principles and recent advancement on cryopreservation of different type of tissues. Organogenesis.

[23] Aitken R.J, Curry B.J (2011). Redox regulation of human sperm function:From the physiological control of sperm capacitation to the etiology of infertility and DNA damage in the germ line. Antioxid. Redox. Signal.

[24] Sharafi M, Borghei-Rad S.M, Hezavehei M, Shahverdi A, Benson J.D (2022). Cryopreservation of semen in domestic animals:A review of current challenges, applications, and prospective strategies. Animals.

[25] Chang F, Zhang B, Liu H, Fan H, Xie R, Li J, Hu Q, Ruan C (2025). Effect of centrifuged chicken egg yolk on the cryopreservation of boar semen. Animals (Basel).

[26] Moussa M, Martinet V, Trimeche A, Tainturier D, Anton M (2002). Low density lipoproteins extracted from hen egg yolk by an easy method:Cryoprotective effect on frozen-thawed bull semen. Theriogenology.

[27] Nisfimawardah L, Ihsan M.N, Susilawati T, Wahjuningsih S, Firmawati A, Sulistyowati C.D, Amalia (2023). Semen quality in the freezing process using various diluents in Saanen goats. In:Developing Modern Livestock Production in Tropical Countries.

[28] Bogdaniuk A, Petrushko M (2022). Seasonal variation of morphometrical characteristics of fresh and cryopreserved Saanen goat sperm. Anim. Reprod.

[29] Suwor F, Kubota S, Nawong S, Thuangsanthia A, Toyra M, Paengkoum P, Ponchunchoovong S (2025). Effects of cryoprotectant combinations on post-thawed sperm quality, biomolecular changes, DNA methylation, and pregnancy rates in Boer goat semen. Vet. Sci.

